# Medicaid Dental Coverage and Preventive Dental Care Use Among Pregnant Adults

**DOI:** 10.1007/s10995-025-04149-9

**Published:** 2025-09-13

**Authors:** Julie C. Reynolds, Tabitha K. Peter, Tessa Heeren, Stephanie E. Lewis, Peter C. Damiano, Xianjin Xie

**Affiliations:** 1 University of Iowa College of Dentistry, Iowa City, IA USA; 2 University of Iowa College of Public Health, Iowa City, USA

**Keywords:** Medicaid dental benefits, Dental care utilization, Pregnancy, PRAMS

## Abstract

**Objectives:**

The aim of this study was to examine the relationship between Medicaid dental coverage for pregnant adults and receipt of a dental cleaning during pregnancy among adults with Medicaid.

**Methods:**

In this cross-sectional study, 2019 Pregnancy Risk Assessment Monitoring System data were used to measure individuals’ receipt of a dental cleaning during pregnancy and in the year prior to pregnancy. The independent variable was the state-level degree of Medicaid dental coverage for pregnant adults as of 2019, ranging from none to extensive. Weighted logistic regression models estimated the odds of having a dental cleaning during pregnancy, both for the full sample and stratified by whether or not a cleaning was received pre-pregnancy.

**Results:**

Approximately one-third (31%) of respondents reported receiving a dental cleaning during pregnancy. In multivariable results, pregnant adults living in states with emergency (OR 0.58, 95% CI 0.41–0.83, *p* = 0.002) or no (OR 0.60, 95% CI 0.41–0.86, *p* = 0.006) dental coverage had significantly lower odds of having a dental cleaning during pregnancy than those living in states with extensive dental coverage. This association was concentrated among people who did not have a dental cleaning pre-pregnancy; those without a cleaning pre-pregnancy who lived in a state with extensive dental coverage had approximately twice the odds or more of having a dental cleaning during pregnancy than those who lived in states with emergency or no dental coverage.

**Conclusions:**

Having dental insurance is critically important to be able to access needed dental care and avoid substantial out-of-pocket costs. This study found that for pregnant adults in Medicaid who who hadn’t had a dental cleaning in the year prior to pregnancy, having extensive dental coverage was significantly associated with much higher odds of having a dental cleaning during pregnancy relative to having emergency or no dental coverage. Our findings, together with the body of evidence on the impact of Medicaid dental coverage on dental care access, underscore the importance of providing comprehensive dental coverage in Medicaid regardless of age and pregnancy status.

## Significance

This study provides important evidence on the role of Medicaid dental coverage in promoting preventive dental care use during pregnancy. Results highlight that pregnant adults in states with more extensive dental coverage were significantly more likely to receive a dental cleaning during pregnancy, especially among those who had not accessed dental care in the year prior to pregnancy. These findings underscore the importance of comprehensive Medicaid dental benefits in reducing barriers to care. They also support policy efforts to expand and standardize dental coverage for pregnant adults across states, reinforcing the importance of oral health care as an integral component of prenatal care.

## Introduction

Dental care use during pregnancy is critically important for several reasons, including that untreated periodontal disease is associated with low birthweight (Castaño-Suárez et al., 2024; Xiong et al., 2007; Xu & Han, 2022) and mothers of young children can transfer cariogenic bacteria to the young child (Chaffee et al., 2014). Additionally, receiving dental care during pregnancy is associated with improved oral health outcomes for young children (Xiao et al., 2019). For these reasons, professional recommendations suggest that pregnant people should receive dental care during pregnancy in order to establish or maintain a dental home and improve their oral health and related behaviors prior to the arrival of their child (National Maternal and Child Oral Health Resource Center, 2012). However, a low proportion of pregnant people receive dental care during pregnancy (Rocha et al., 2018), and pregnant people enrolled in Medicaid are significantly less likely to see a dentist during pregnancy than those with private insurance (Gaffield et al., 2001; Kranz & Estrada-Darley, 2022; Lee et al., 2022a, 2022b; Robison et al., 2021).

Access to dental coverage is particularly crucial for people with low income because out-of-pocket costs for dental care pose substantial challenges to receiving needed care. Yet, unlike dental coverage for children, dental coverage for pregnant adults in Medicaid is not mandatory under federal law (Silverman, 2012). States can therefore dictate the extent of Medicaid dental coverage for pregnant adults, which ranges from extensive coverage to none at all. As of 2019, 2 states provided no dental coverage for pregnant adults, 7 provided coverage for emergency services only, 19 provided limited coverage, and 22 provided extensive coverage (Eke et al., 2019). Several studies have found that higher Medicaid dental coverage leads to higher dental care utilization for non-pregnant adults (Abdus & Decker, 2019; Elani et al., 2020; Singhal et al., 2017). However, very few studies have examined the relationship between Medicaid dental coverage for pregnant adults and dental care use during pregnancy. One study found that higher coverage was associated with higher preventive dental visits before and during pregnancy, as well as visits for a dental problem during pregnancy, after adjusting for other factors (Lee et al., 2022a, 2022b). Additionally, a related study in Virginia found that increasing Medicaid dental coverage for pregnant people resulted in higher dental utilization during pregnancy post-policy change (Naavaal & Harless, 2022).

Given that dental coverage is a critical predictor of access to dental care, and the importance of adequate access to care during pregnancy, more evidence is needed about the impacts of Medicaid dental coverage for pregnant adults. The aim of this study was to examine the relationship between state-level Medicaid dental coverage for pregnant adults and individual-level utilization of preventive care during pregnancy among Medicaid-enrolled pregnant adults.

## Materials and methods

The main data source for this cross-sectional study was the Pregnancy Risk Assessment Monitoring Survey (PRAMS), a U.S. survey of people who have had a recent live birth. It is a mixed-mode—mail and telephone—survey that is administered by state, territorial, tribal, or local health departments using a standard data collection protocol in partnership with the Centers for Disease Control and Prevention (Shulman et al., 2018). States must meet a 50% response rate or higher to be included in the national dataset. PRAMS questionnaires are composed of Core Questions that are required for all states to include, and Standard Questions that are optional.

The study sample was composed of respondents to the 2019 (Phase 8) PRAMS survey who were enrolled in Medicaid during pregnancy. Respondents were included in the sample if they reported Medicaid as their only source of insurance for prenatal care. The 2019 PRAMS data included 40 states with available data that met the 50% response rate criteria for inclusion. Respondents were excluded if they were under age 21 as most of these respondents would have been eligible for comprehensive dental coverage per federal requirements under the Early and Periodic Screening, Diagnosis, and Treatment guidelines. PRAMS analysis weights—which adjust for design, nonresponse, and noncoverage—were used to obtain population-based estimates. Complete case analysis was used, and sample subsetting for Medicaid-enrolled pregnant adults was done in accordance with PRAMS data user guidance to maintain appropriate weights. The final analytical sample included 11,915 unweighted and 611,755 weighted subjects, which included those that met inclusion criteria and had complete data on all study variables.

The dependent variable was having had a dental cleaning during the most recent pregnancy (Y/N). Individual-level covariates included age in years, race/ethnicity, educational attainment, marital status, use of WIC during pregnancy, and health issues during pregnancy. Health issues during pregnancy was defined as yes if the respondent reported that they had diabetes, high blood pressure, and/or depression during pregnancy, and no if none. These covariates were selected based on evidence from prior work or hypothesized relationship with dental care use (Singhal et al., 2014). Other variables considered but not used due to data missingness or correlation with other variables included urbanicity, household income, and the number of prenatal care visits. 

The primary independent variable was the state’s level of Medicaid dental coverage for pregnant adults as of September 2019, which was sourced from a report by the Children’s Dental Health Project (Eke et al., 2019). The variable was categorized as *Extensive* (defined as coverage of more than 100 diagnostic, preventive, and restorative procedures with an annual maximum of at least $1000), *Limited* (defined as coverage of fewer than 100 diagnostic preventive, and restorative procedures, with an annual maximum of $1000 or less), coverage for *Emergency* dental services only (defined as coverage for relieving pain and/or infection under emergency circumstances), and *No dental coverage* (hereafter referred to as None). Among the final 40 states in this study, 19 provided extensive dental coverage, 15 provided limited coverage, 4 provided emergency coverage, and 2 states provided none.

Other state-level characteristics were considered for inclusion based on evidence of association with dental care use. Dentist supply was sourced from the Health Resources and Services Administration and defined as the number of dentists in the state per 100,000 population as of 2018–2019 (Bureau of Health Workforce, 2022). We also considered including the Medicaid eligibility threshold for pregnant adults as well as the proportion of dentists in the state enrolled as Medicaid providers. However, both were ultimately not included due to their correlation with other state variables or indicators of poorer model fit. Seven candidate models were compared using BIC and condition number to assess fit and multicollinearity, respectively (Liao & Valliant, 2012; Neath & Cavanaugh, 2012). The final model was selected based on optimizing model fit and minimizing multicollinearity. As a sensitivity analysis, we included both variables (dentist Medicaid enrollment and income eligibility threshold for pregnant adults) in bivariate analyses among the full sample and neither was significantly associated with our outcome, which confirmed the appropriateness to omit them from the final model.

Weighted bivariate Chi-square tests with the Rao-Scott correction to account for large sample size were used for categorical variables, and continuous variables were tested using Student’s t-test adapted to complex survey samples; both were calculated via the “survey” and “srvyr” R packages (R version 4.2). A type 1 error rate threshold of 0.05 was used to assess the degree of evidence from these tests. Due to multiple levels of covariates (state and individual), hierarchical logistic regression was initially used to account for within-state correlation. However, the results of our hierarchically structured model indicated that the variation within each state was near zero, having a value of only 0.09. Such small among-state variance yielded a near-zero estimate of intra-class (within-state) correlation, thus providing a lack of evidence that the data followed a hierarchical structure. For these reasons, we did not pursue a hierarchical model and instead utilized standard logistic regression. Models were generated for the full sample and stratified by whether or not a cleaning was received in the 12 months prior to pregnancy. All logistic regression models adjusted for individual-level covariates. For continuous independent variables, odds ratios indicate the odds associated with a 10-percentage point or unit increase. This study was deemed not human subjects research by the University of Iowa Institutional Review Board as all data were deidentified.

## Results

The final analytical sample included 11,862 unweighted and 609,152 weighted subjects, which included those that met inclusion criteria and had complete data on all study variables. The highest proportions of respondents had completed high school (41%), were white (42%), were not married (66%), had used WIC during pregnancy (64%), and did not have health issues during pregnancy (63%) (Table 1). Nearly half (49%) lived in states that provided extensive dental coverage to pregnant adults in Medicaid, whereas 36% lived in states with limited coverage, 8.4% with emergency coverage, and 6.1% with no dental coverage. State dentist workforce supply was 59 dentists per 100,000 population, on average, for respondents in the full sample.

**Table 1:** Descriptive characteristics of study subjects by receipt of a dental cleaning during pregnancy, Pregnancy Risk Assessment Monitoring System, 2019

Characteristic	Weighted *n* = 609,152 Mean (range) or *n* (%)	Dental cleaning during pregnancy Mean (SD) or *n*(%)		*p* value
		Yes (*n* = 186,462, 31%)	No (*n* = 422,690, 69%)	
Individual characteristics				
Age	28.7 (21.0, 51.0)	28.9 (5.3)	28.7 (5.2)	0.10
Educational attainment				< 0.001
Less than 8th grade	20,839 (3.4%)	6,524 (31%)	14,315 (69%)	
Some high school	85,173 (14%)	22,558 (26%)	62,614 (74%)	
Completed high school	251,520 (41%)	76,605 (30%)	174,915 (70%)	
Some college	195,237 (32%)	57,581 (29%)	137,656 (71%)	
4-year degree or higher	56,384 (9.3%)	23,195 (41%)	33,190 (59%)	
Race/ethnicity				< 0.001
American Indian/Alaska Native	5,369 (0.9%)	1,750 (33%)	3,619 (67%)	
Asian	20,909 (3.4%)	6,516 (31%)	14,393 (69%)	
Black	163,487 (27%)	47,532 (29%)	115,956 (71%)	
Hispanic	141,762 (23%)	52,015 (37%)	89,747 (63%)	
White	258,416 (42%)	70,467 (27%)	187,949 (73%)	
Other race or multiracial	19,208 (3.2%)	8,182 (43%)	11,027 (57%)	
Marital status				0.069
Married	207,304 (34%)	67,083 (32%)	140,221 (68%)	
Not married	401,848 (66%)	119,380 (30%)	282,469 (70%)	
Used WIC during pregnancy				0.056
Yes	388,113 (64%)	122,701 (32%)	265,413 (68%)	
No	221,039 (36%)	63,762 (29%)	157,277 (71%)	
Health issues during pregnancy				0.003
Yes	225,980 (37%)	63,130 (28%)	162,850 (72%)	
No	383,172 (63%)	123,332 (32%)	259,840 (68%)	
Received dental cleaning before pregnancy				< 0.001
Yes	143,791 (24%)	86,446 (60%)	57,345 (40%)	
No	465,361 (76%)	100,016 (21%)	365,345 (79%)	
State characteristics				
Medicaid dental coverage for pregnant people				< 0.001
Extensive	299,556 (49%)	108,020 (36%)	191,536 (64%)	
Limited	221,638 (36%)	62,883 (28%)	158,755 (72%)	
Emergency	50,885 (8.4%)	9,347 (18%)	41,537 (82%)	
None	37,074 (6.1%)	6,212 (17%)	30,862 (83%)	
Dentist workforce supply	58 (40, 82)	62 (11)	58 (11)	< 0.001

Approximately one-third (31%) of pregnant people had a dental cleaning during pregnancy, and 24% had a dental cleaning in the year before pregnancy (Table 1). Among those that had a cleaning pre-pregnancy, 60% had one during pregnancy. Among those that did not have a cleaning pre-pregnancy, 21% had one during pregnancy.

Bivariate results show evidence that receipt of a dental cleaning during pregnancy was associated with educational attainment, race/ethnicity, use of WIC during pregnancy, health issues during pregnancy, Medicaid dental coverage for pregnant adults, dentist workforce supply, and receipt of a dental cleaning before pregnancy (Table 1). Those who were most likely to receive a dental cleaning during pregnancy were respondents with a 4-year degree or higher, people who were multiracial or some other race, used WIC during pregnancy, had a dental cleaning before pregnancy, and did not have a health issue during pregnancy. Among respondents living in states with Extensive dental coverage, 36% had a dental cleaning during pregnancy, compared to 28% of those in states with Limited dental coverage, 18% of those in states with Emergency, and 17% of those in states with None. Respondents who had a dental cleaning during pregnancy lived in states with a significantly higher supply of dentists, on average, relative to those who did not have a dental cleaning during pregnancy.

Multivariable results indicated that after adjusting for covariates, pregnant adults living in states with emergency (OR 0.58, 95% CI 0.41–0.83, *p* = 0.002) or no (OR 0.60, 95% CI 0.41–0.86, *p* = 0.006) dental coverage had 40–42% lower odds of having a dental cleaning during pregnancy than those living in states with extensive dental coverage (Table 2). Odds of having a dental cleaning during pregnancy were not significantly different between those living in states with Extensive vs. Limited dental coverage. Stratified model results showed that this association was concentrated among people who did not have a cleaning pre-pregnancy (Table 2). Among people who had a cleaning prior to pregnancy, there was no association between dental coverage and receipt of a cleaning during pregnancy. However, those who didn’t have a cleaning pre-pregnancy and lived in a state with extensive dental coverage had approximately twice the odds or more of having a dental cleaning during pregnancy than those who lived in states with emergency (OR 0.51, 95% CI 0.32–0.80, *p* = 0.004) or no (OR 0.40, 95% CI 0.24–0.66, *p* < 0.001) dental coverage.

**Table 2:** Multivariable logistic regression results estimating the odds of a dental cleaning during pregnancy among pregnant adults with Medicaid, Pregnancy Risk Assessment Monitoring System, 2019

Characteristic	Full sample (weighted *n* = 609,152)			Received a dental cleaning before pregnancy (weighted *n* = 143,791)			Did not receive a dental cleaning before pregnancy (weighted *n* = 465,361)		
	OR	95% CI	p value	OR	95% CI	p value	OR	95% CI	p value
Age	0.97	0.85, 1.12	0.7	0.90	0.70, 1.15	0.4	1.02	0.86, 1.20	0.8
Marital status									
Married	–	–		–	–		–	–	
Unmarried	0.96	0.82, 1.12	0.6	1.02	0.78, 1.34	0.9	0.94	0.78, 1.13	0.5
Educational attainment									
Less than 8th grade	0.72	0.47, 1.12	0.14	1.12	0.44, 2.90	0.8	0.68	0.41, 1.12	0.13
Some high school	0.64	0.47, 0.86	0.003	0.65	0.38, 1.11	0.12	0.65	0.45, 0.94	0.021
Completed high school	0.75	0.58, 0.97	0.026	0.85	0.56, 1.28	0.4	0.73	0.53, 1.00	0.050
Some college	0.62	0.48, 0.80	< 0.001	0.60	0.40, 0.90	0.013	0.65	0.48, 0.89	0.008
4-year degree or higher	–	–		–	–		–	–	
Race/ethnicity									
American Indian/Alaska Native	1.41	1.00, 1.98	0.051	1.27	0.62, 2.57	0.5	1.47	0.99, 2.17	0.054
Asian	1.02	0.73, 1.43	0.9	1.27	0.62, 2.59	0.5	0.97	0.65, 1.43	0.9
Black	1.17	0.98, 1.40	0.080	0.97	0.72, 1.32	0.9	1.31	1.05, 1.63	0.019
Hispanic	1.63	1.34, 1.98	< 0.001	1.37	0.96, 1.97	0.086	1.74	1.39, 2.19	< 0.001
Other race or Multiracial	2.07	1.40, 3.06	< 0.001	1.35	0.70, 2.59	0.4	2.36	1.50, 3.70	< 0.001
White	–	–		–	–		–	–	
Use of WIC during pregnancy									
Yes	1.20	1.03, 1.38	0.016	0.99	0.77, 1.28	> 0.9	1.31	1.09, 1.57	0.003
No	–	–		–	–		–	–	
Health issues during pregnancy									
Yes	0.83	0.72, 0.96	0.012	0.81	0.63, 1.05	0.11	0.85	0.71, 1.02	0.076
No	–	–		–	–		–	–	
Recived dental cleaning before pregnancy									
Yes	5.66	4.85, 6.60	< 0.001						
No	–	–		–	–		–	–	
Medicaid dental coverage for pregnant people									
Extensive	–	–		–	–		–	–	
Limited	0.87	0.73, 1.04	0.13	0.85	0.63, 1.14	0.3	0.89	0.72, 1.10	0.3
Emergency	0.58	0.41, 0.83	0.002	0.80	0.42, 1.51	0.5	0.51	0.32, 0.80	0.004
None	0.60	0.41, 0.86	0.006	1.27	0.59, 2.75	0.5	0.40	0.24, 0.66	< 0.001
Dentist workforce supply	1.18	1.10, 1.27	< 0.001	1.10	0.97, 1.25	0.14	1.22	1.12, 1.33	< 0.001

## Discussion

In this cross-sectional study using 2019 PRAMS data from 40 U.S. states, we found that after adjusting for other factors likely to affect dental care access, higher state Medicaid dental coverage was significantly associated with greater odds of receiving a dental cleaning during pregnancy. Pregnant adults in states with extensive dental coverage in Medicaid had significantly higher odds of having a dental cleaning during pregnancy than those living in states with emergency-only or no dental coverage, and this association was concentrated among people who had not had a dental cleaning in the year prior to pregnancy. Our findings are consistent with a similar study that used 2014–2015 PRAMS data from 26 states to explore a similar research question (Lee et al., 2022a, 2022b). The study used three categories of Medicaid dental coverage—No coverage for cleaning, Limited coverage (dental cleaning and fillings), and Extended coverage (more than dental cleaning and fillings)—and found that pregnant adults in states with Limited or Extended coverage had a significantly higher adjusted prevalence of having a dental cleaning during pregnancy relative to those in states without coverage for a cleaning. The study also found that among adults with a dental problem during pregnancy, those living in states with Limited or Extended coverage had significantly higher prevalence of having dental visit for that problem relative to those in states with no coverage. Ours and the Lee et al. study are cross-sectional, which have inherent limitations in the inability to account for other potential state characteristics that may influence dental care access. One study has used a stronger longitudinal quasi-experimental study design to compare dental utilization among pregnant adults before and after the state of Virginia increased dental coverage for this population (Naavaal & Harless, 2022). Naavaal et al. used six cycles of Virginia PRAMS data to examine the effect of this policy change, which increased dental coverage in 2015 from emergency-only to extensive. They found that compared to pre-policy change, as of 2019 there was a 17 percentage point increase in the probability of dental utilization among pregnant people in Medicaid relative to the change in probability among those with private insurance. Our results are also consistent with evidence demonstrating that increasing adult Medicaid dental coverage is associated with improved utilization of dental care for non-pregnant adults (Nasseh & Vujicic, 2013; Singhal et al., 2015) as well as spillover effects of these policies on dental utilization among children (Khouja et al., 2020; Lipton, 2021; Lipton et al., 2021).

We found that there was a higher rate of having a dental cleaning during pregnancy (31%) than in the year before pregnancy (24%) among this study population, and that the association between Medicaid dental coverage and receipt of a dental cleaning during pregnancy was concentrated among those who hadn’t had a cleaning prior to pregnancy. Most states have a higher income eligibility limit for pregnant people than non-pregnant adults. As a result, a person with low income who has the same household income before, during, and after pregnancy is much more likely to be eligible for Medicaid during pregnancy than before or after. One study found that among people who were enrolled in Medicaid during pregnancy, 27% were uninsured before pregnancy, and 22% became uninsured within 6 months postpartum (Johnston et al., 2021). Thus, the period of pregnancy is a critical window to be able to provide comprehensive coverage for dental care given that many beneficiaries may not have such coverage before or after. Our results demonstrating a higher proportion receiving a dental cleaning during pregnancy may reflect these variations in eligibility, though more research is needed to understand the drivers of these patterns. Recent federal efforts have led nearly all states to extend continuous coverage to 12 months postpartum; however, the degree of dental coverage available during and after pregnancy continues to vary. As of October 2022, all 50 states had some degree of dental coverage for pregnant adults; however, the degree of coverage ranged from emergency-only in five states, limited in 13 states, and extensive in 32 states and DC (National Academy of State Health, 2022).

Limitations to this study include the cross-sectional design and survey data source. Results of cross-sectional studies on associations of dental care access and state-level policies are inherently less robust due to the inability to account for unobservable sources of confounding. Therefore, causality cannot be inferred and future studies could utilize quasi-experimental study designs, where possible, that leverage state policy changes expanding dental coverage coverage for pregnant people. Self-reported survey data have inherent risk of bias, including recall bias and self-desirability bias, which may affect the validity of respondents’ reports of dental care utilization during and before pregnancy. A previous study found good-excellent agreement between self-report and dental chart sources for receipt of a dental cleaning, but this study is dated and a similar question has not been examined for pregnant people in particular (Gilbert et al., 2003).

Our findings, together with the body of evidence on the impact of Medicaid dental coverage on dental care access, underscore the importance of providing comprehensive dental coverage in Medicaid regardless of age and pregnancy status. States are required to provide comprehensive dental coverage for children, but are not required to provide it for adults over age 21. For people with low income in particular, having insurance is critically important to be able to access needed dental care and avoid substantial out-of-pocket costs. Provision of comprehensive dental coverage during pregnancy and during the post partum period is likely to have long-term positive impacts on oral health of the pregnant person and, subsequently, their child.
